# The real-world safety profile of sodium-glucose co-transporter-2 inhibitors among older adults (≥ 75 years): a retrospective, pharmacovigilance study

**DOI:** 10.1186/s12933-023-01743-5

**Published:** 2023-01-24

**Authors:** Adam Goldman, Boris Fishman, Gilad Twig, Emanuel Raschi, Tali Cukierman-Yaffe, Yonatan moshkovits, Alon Pomerantz, Ilan Ben-Zvi, Rachel Dankner, Elad Maor

**Affiliations:** 1 Department of Epidemiology and Preventive Medicine, School of Public Health, Sackler School of Medicine, Tel Aviv University, Tel Aviv, Israel; 2 Leviev Heart Center, Sheba Medical Center, Ramat-Gan, Israel; 3 Department of Internal Medicine, Sheba Medical Center, Ramat-Gan, Israel; 4 The Talpiot Sheba Medical Leadership Program, Ramat-Gan, Israel; 5 Sackler Faculty of Medicine, Tel-Aviv University, Tel-Aviv, Israel; 6 Division of Endocrinology & Metabolism, Sheba Medical Center, Ramat-Gan, Israel; 7 Pharmacology Unit, Department of Medical and Surgical Sciences, Alma Mater Studiorum, University of Bologna, Bologna, Italy; 8 The Gertner Institute for Epidemiology and Health Policy Research, Sheba Medical Center, Ramat-Gan, Israel

**Keywords:** Sodium-glucose co-transporter-2 inhibitors, Older adults, Diabetes, Heart failure, Diabetic ketoacidosis, Fournier gangrene

## Abstract

**Background:**

As indications for sodium-glucose co-transporter-2 inhibitors (SGLT2i) are expanding, a growing number of older adults have become candidates for treatment. We studied the safety profile of SGLT2i among older adults.

**Methods:**

A retrospective, pharmacovigilance study of the FDA’s global database of safety reports. To assess reporting of pre-specified adverse events following SGLT2i among adults (< 75 years) and older adults (≥ 75), we performed a disproportionality analysis using the sex-adjusted reporting odds ratio (adj.ROR).

**Results:**

We identified safety reports of 129,795 patients who received non-insulin anti-diabetic drugs (NIAD), including 24,253 who were treated with SGLT2i (median age 60 [IQR: 51–68] years, 2,339 [9.6%] aged ≥ 75 years). Compared to other NIAD, SGLT2i were significantly associated with amputations (adj.ROR = 355.1 [95%CI: 258.8 − 487.3] vs adj.ROR = 250.2 [79.3 − 789.5]), Fournier gangrene (adj.ROR = 45.0 [34.5 − 58.8] vs adj.ROR = 88.0 [27.0 − 286.6]), diabetic ketoacidosis (adj.ROR = 32.3 [30.0 − 34.8] vs adj.ROR = 23.3 [19.2 − 28.3]), genitourinary infections (adj.ROR = 10.3 [9.4 − 11.2] vs adj.ROR = 8.6 [7.2 − 10.3]), nocturia (adj.ROR = 5.5 [3.7 − 8.2] vs adj.ROR = 6.7 [2.8 − 15.7]), dehydration (adj.ROR = 2.5 [2.3 − 2.8] vs adj.ROR = 2.6 [2.1 − 3.3]), and fractures (adj.ROR = 1.7 [1.4 − 2.1] vs adj.ROR = 1.5 [1.02 − 2.1]) in both adults and older adults, respectively. None of these safety signals was significantly greater in older adults (P_interaction_ threshold of 0.05). Acute kidney injury was associated with SGLT2i in adults (adj.ROR = 1.97 [1.85 − 2.09]) but not in older adults (adj.ROR = 0.71 [0.59 − 0.84]). Falls, hypotension, and syncope were not associated with SGLT2i among either adults or older adults.

**Conclusion:**

In this global post-marketing study, none of the adverse events was reported more frequently among older adults. Our findings provide reassurance regarding SGLT2i treatment in older adults, although careful monitoring is warranted.

**Supplementary Information:**

The online version contains supplementary material available at 10.1186/s12933-023-01743-5.

## Introduction

Sodium-glucose co-transporter-2 inhibitors (SGLT2i) have changed the treatment landscape of type II diabetes mellitus (T2DM) and demonstrated efficacy in reducing blood glucose and diabetic-related complications [1, 2]. SGLT2i have been shown to slow the progression of diabetic kidney disease and reduce heart failure (HF) hospitalizations and cardiovascular death [3, 4]. Therefore, clinical trials were designed to assess their role in patients with HF with reduced ejection fraction and found a lower risk of cardiovascular death or hospitalizations irrespective of diabetes status [5, 6]. Recently, empagliflozin was the first medication to exhibit a significant beneficial effect in patients with HF with preserved ejection fraction, which was consistent across all age groups [7, 8]. As indications for SGLT2i treatment are expanding, a growing number of older adults have become candidates for treatment, prompting a clinical need to assess the treatment safety in this population.

SGLT2i were well-tolerated in clinical trials, with genitourinary infections being the most common adverse event (AE) [1, 2]. Additional potential therapy-related AEs are an ongoing source of concern, including diabetic ketoacidosis (DKA), acute kidney injury (AKI), amputations, fractures, hypoglycemia, and Fournier gangrene [9–13]. Older adults might be at a greater risk of developing treatment-related complications due to frailty, polypharmacy, and different pharmacokinetic and pharmacodynamic properties [14]. However, safety data in this population are limited and mainly derived from clinical trials, in which the geriatric population was underrepresented. Moreover, clinical trials may not represent the real-world population and are usually underpowered to detect rare AEs, particularly in sub-populations. Therefore, post-marketing surveillance programs may provide complementary information. Herein, we aim to characterize the safety profile of SGLT2i among older adults (age ≥ 75 years) in real-world settings using the FDA adverse events reporting system (FAERS), a global data repository of post-marketing safety reports.

## Methods

### Data sources and study design

An observational, retrospective, pharmacovigilance study using the FAERS, a global post-marketing surveillance program [15, 16]. The FAERS include voluntary and mandatory safety reports submitted by healthcare professionals, consumers, and manufacturers. The database was screened for reports of non-insulin anti-diabetic drugs (NIAD) as the primary suspects of a given AE between July 1, 2014, and June 30, 2021. Patients who reported SGLT2i (canagliflozin, dapagliflozin, empagliflozin, or ertugliflozin) as the primary suspect were defined as the exposure group, while all other NIAD were the comparator group. We excluded patients below 18 years of age, patients receiving the treatment for indications other than T2DM, and patients who reported concomitant treatment with insulin. In case multiple reports of the same event were detected, only the latest case version of every event was retained, as recommended by the FDA. We further applied an algorithm to eliminate suspected duplicate reports of the same drug-AE pair with different case numbers, by screening for identical values in four key fields: age, sex, event date, and country of occurrence [17]. Patients aged 18–75 years were defined as adults and patients older than 75 years as older adults.

### Endpoints

AEs in the FAERS are coded at the preferred-term level of the Medical Dictionary for Regulatory Activities (MedDRA) classification [18]. Due to a large number of preferred terms (~ 25,000) and their lack of specificity, Standardized MedDRA Queries (SMQs) were developed. SMQs are standard sets of MedDRA terms that are related to the same medical condition, thereby facilitating data retrieval and signal detection [18, 19].

In this study, 13 pre-specified AEs of interest were selected based on previous reports [1, 2, 5, 6]. AE definitions included all terms in the corresponding SMQ (MedDRA version 25.1), in case it was available, with a manual validation by the author (Additional file 1: Table S1). Otherwise, the definitions of the AEs were based on high-level terms in the MedDRA hierarchy [19].

### Statistical analysis

We performed a disproportionality analysis to compare the proportion of specific AEs following SGLT2i (cases) with the corresponding proportion of other NIAD in the entire database (non-cases), also known as case/non-case analysis [17]. We used a restricted comparator group of NIAD other than SGLT2i, also known as disproportionality by therapeutic area [20], in order to generate groups sharing common characteristics, thereby minimizing confounding by indication. We used the reporting odds ratio (ROR) and the lower bound of the information component 95% credibility interval (IC_025_), which are well-validated measures to detect signals of disproportionate reporting in post-marketing passive-surveillance databases [21–23]. These measures evaluate whether a drug-AE pair is reported higher-than-expected. The expected number is the AE occurrence by any other drug in the reference group. Reporting odds ratio, a frequentist measure, is the pharmacovigilance equivalent of the odds ratio, and therefore easily communicated to non-statisticians. Crude RORs were calculated with a shrinkage transformation as described by Noren et al. [24] and the sex-adjusted RORs (adj.ROR) were calculated by logistic regression models. IC_025_, a Bayesian measure that also accounts for disproportionate reporting, has been shown to reduce false positive results when a small number of cases is reported [21, 22]. A lower bound of the ROR 95% confidence interval (CI) greater than one and a positive IC_025_ value are the traditional thresholds for signal detection, also used in this study.

We added an interaction term to the model to estimate for interaction between SGLT2i treatment and age group (i.e., adults and older adults). To evaluate whether the SGLT2i type affects the observed associations (drug effect rather than class effect), we stratified the analysis by SGLT2i product. In addition, we conducted a stratified analysis by event year to study the reporting pattern of SGLT2i-related AKI over the years.

Continuous variables were non-normally distributed, as reflected by the Shapiro–Wilk test and visual inspection of quantile–quantile plots and histograms, and therefore they were presented by median [interquartile range (IQR)]. Categorical data were compared by the chi-square test or the Fisher’s exact test. All tests were two-sided with a significance level of p-value < 0.05. Data processing and statistical analyses were performed in R statistical software (R Foundation for Statistical Computing).

## Results

### Characteristics of patients

**Fig. 1:**
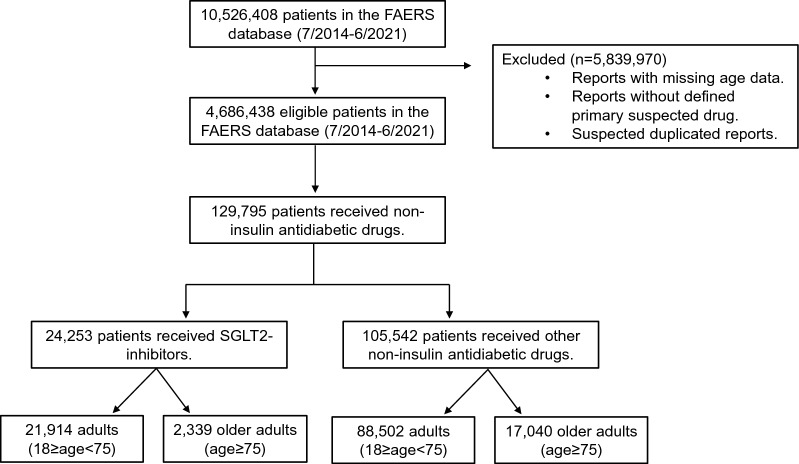
Study flowchart. FAERS- FDA adverse event reporting system

The FAERS database included safety reports of 4,686,438 eligible patients. We identified safety reports of 129,795 patients who reported NIAD as the primary suspected drug, of whom 24,253 SGLT2i recipients (Fig. 1). The median [IQR] age of patients treated with SGLT2i and other NIAD was 60 [51–68] years and 64 [54–73] years, respectively, but a smaller proportion of SGLT2i recipients were older adults (n = 2,339/24,253 [9.6%], n = 17,040/105,542 [16.1%], p < 0.001) (Additional file 2: Table S2). The median age of patients treated with other NIAD was 60 [52–67] years for adults and 79 [77–83] years for older adults. The proportion of females was 45.3% among SGLT2i recipients and 52.1% among recipients of other NIAD. 56% of SGLT2i reports were submitted by healthcare providers, mostly from the Americas (n = 16,427/23,656 [69%]), followed by Europe (n = 3972/23,656 [17%]) and Asia (2758/23,656 [12%]) (Table 1). Canagliflozin had the greatest number of reports in the FAERS (n = 10,872), followed by empagliflozin (n = 8112), dapagliflozin (n = 5099), and ertugliflozin (n = 170). The main NIAD medications other than SGLT2i in the FAERS were glucagon-like peptide-1 receptor agonists (44,385 reports [42.1% of all other NIAD reports]), thiazolidinediones (25,837 [24.4%]), metformin (22,788 [21.6%]), dipeptidyl peptidase-4 inhibitors (10,281 [9.7%]), and insulin secretagogues (2,381 [2.3%]). A higher proportion of older SGLT2i recipients were treated concomitantly with diuretics, angiotensin-converting enzyme (ACE) inhibitors, angiotensin receptor blockers (ARBs), beta-blockers, statins, steroids, and PPI, as compared to younger recipients (all p values < 0.001, Table 1).

**Table 1:** Demographic and clinical characteristics of adults (< 75 years) and older adults (≥ 75 years) treated with SGLT2-inhibitors in the FAERS

	ALL (24,253)	Adults (21,914)	Older adults (2339)
*Region*			
Americas^a^	16,427/23,647 (69.4)	15,134/21,338 (70.9)	1,284/2,309 (55.3)
Europe	3,972/23,647 (16.7)	3,511/21,338 (16.4)	461/2,309 (19.9)
Asia	2,758/23,647 (11.7)	2,262/21,338 (10.6)	496/2,309(21.4)
Africa	61/23,647 (0.3)	59/21,338 (0.3)	2/2,309 (0.1)
Australia	438/23,647 (1.9)	372/21,338 (1.8)	66/2,309 (3.3)
*Reporter*			
Health professional	12,882/23,100 (55.8)	11,412/20,878 (54.7)	1,470/2,222 (66.2)
Patient/lawyer	10,218/23,100 (44.2)	9,466/20,878 (43.2)	752/2,222 (33.8)
*Year of report*			
2014^b^	652/24,253 (2.7)	603/21,914 (2.8)	49/2,339 (2.1)
2015	3,465/24,253 (14.3)	3,157/21,914 (14.4)	308/2,339 (13.2)
2016	2,747/24,253 (11.3)	2,490/21,914 (11.4)	257/2,339 (11.0)
2017	3,057/24,253 (12.6)	2,775/21,914 (12.7)	282/2,339 (12.1)
2018	5,426/24,253 (22.4)	5,044/21,914 (23.0)	382/2,339 (16.3)
2019	4,038/24,253 (16.6)	3,619/21,914 (16.5)	419/2,339 (17.9)
2020	3,201/24,253 (13.2)	2,790/21,914 (12.7)	411/2,339 (17.6)
2021	1,667/24,253 (6.9)	1,436/21,914 (6.6)	231/2,339 (9.9)
*Medication*			
Canagliflozin	10,872/24,253 (44.8)	10,115/21,914 (46.2)	757/2,339 (32.4)
Dapagliflozin	5,099/24,253 (21.0)	4,560/21,914 (20.8)	539/2,339 (23.0)
Empagliflozin	8,112/24,253 (33.4)	7,078/21,914 (32.3)	1,034/2,339 (44.2)
Ertugliflozin	170/24,253 (0.7)	161/21,914 (0.7)	9/2,339 (0.4)
*Age*, years			
Median (IQR)	60 (51–68)	58 (50–65)	79 (76–82)
*Sex*			
Females	10,885/24,006 (45.3)	9,819/21,695 (45.3)	1,066/2,311 (46.1)
Males	13,121/24,006 (54.7)	11,876/21,695 (54.7)	1,245/2,311 (53.9)
*Concomitant drugs*			
Metformin	9,480 (39.1)	8,655 (39.5)	825 (35.3)
GLP-1-agonists / DPP-4 inhibitors	4,895 (20.2)	4,274 (19.5)	621 (26.6)
Sulfonylureas	2,718 (11.2)	2,359 (10.8)	359 (15.4)
ACE inhibitors / ARBs	4,208 (17.4)	3,686 (16.8)	522 (22.3)
Diuretics^c^	1,529 (6.3)	1,280 (5.8)	249 (10.7)
Beta-blockers	1,697 (7.0)	1,422 (6.5)	275 (11.8)
Steroids	541 (2.2)	454 (2.1)	87 (3.7)
Stains	4,051 (16.7)	3,530 (16.1)	521 (22.3)
PPI	1,518 (6.3)	1,269 (5.8)	249 (10.7)

Values are n/N (%) unless otherwise specified. a. Americas category includes north and south America; Out of 16,427 recipients of SGLT2-inhibitors from the Americas, 15,158 were from the United States (14,022 adults and 1,136 older adults). b. Only reports from July to December were included. c. Including loop diuretics, thiazides, and aldosterone antagonists. ACE inhibitors- angiotensin-converting enzyme inhibitors; ARBs- angiotensin II receptor blockers; DPP-4- dipeptidyl peptidase-4; FAERS- FDA adverse event reporting system; GLP1- glucagon-like peptide 1; IQR- interquartile range; PPI- proton pump inhibitors; SGLT2- sodium-glucose co-transporter-2

### SGLT2i-related AEs

**Fig. 2:**
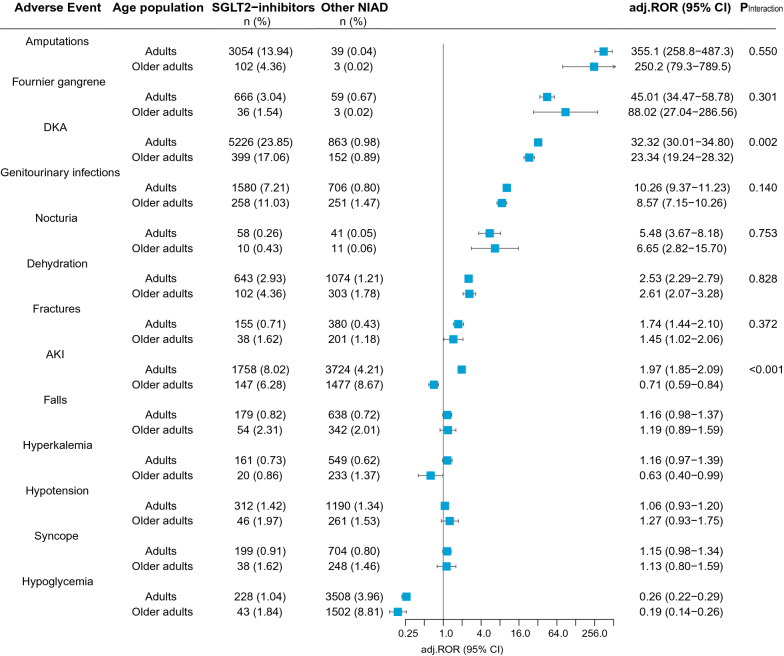
SGLT2-inhibitors related adverse events among adults and older adults. Disproportionality analysis of SGLT2-inhibitors-related adverse events as compared to other non-insulin antidiabetic drugs from the full database. A lower limit of the adj.ROR 95% CI above 1 is the conventional threshold for significant signal detection. Adults are patients aged 18–75, older adults are 75 years or older. adj.ROR- Sex-adjusted reporting odds ratio; AKI- Acute kidney injury; CI- Confidence interval; DKA- Diabetic ketoacidosis; NIAD- non-insulin antidiabetic drugs; SGLT2- Sodium-glucose co-transporter 2

Compared to other NIAD, SGLT2i were significantly associated with the following pre-specified AEs in both adults and older adults after sex adjustment: amputations (n = 3054 [13.9%], adj.ROR = 355.1 [95% CI: 258.8 − 487.3] vs. n = 102 [4.4%], adj.ROR = 250.2 [79.3 − 789.5]), Fournier gangrene (n = 666 [3.0%], adj.ROR = 45.01 [34.47 − 58.78] vs. n = 36 [1.5%], adj.ROR = 88.02 [27.04 − 286.56]), DKA (n = 5226 [23.9%], adj.ROR = 32.32 [30.01 − 34.80] vs. n = 399 [17.1%], adj.ROR = 23.34 [19.24 − 28.32]), genitourinary infections (n = 1580 [7.2%], adj.ROR = 10.26 [9.37 − 11.23] vs. n = 258 [11.0%], adj.ROR = 8.57 [7.15 − 10.26]), nocturia (n = 58 [0.3%], adj.ROR = 5.48 [3.67 − 8.18] vs. n = 10 [0.4%], adj.ROR = 6.65 [2.82 − 15.70]), dehydration (n = 643 [2.9%], adj.ROR = 2.53 [2.29 − 2.79] vs. n = 102 [4.4%], adj.ROR = 2.61 [2.07 − 3.28]), and fractures (n = 155 [0.7%], adj.ROR = 1.74 [1.44 − 2.10] vs. n = 38 [1.6%], adj.ROR = 1.45 [1.02 − 2.06]) (Fig. 2). The IC_025_ and the crude ROR with shrinkage transformation of all of these safety signals were statistically significant apart from fractures in older adults (IC_025_ = -− -0.10, ROR = 1.38 [0.98–1.96]) (Additional file 2: Table S3).

SGLT2i treatment was associated with AKI in adults (adj.ROR = 1.97 [1.85–2.09]) but not in older adults (adj.ROR = 0.71 [0.59–0.84]) (Fig. 2). Falls, hyperkalemia, hypotension, syncope, and hypoglycemia were not significantly over-reported among SGLT2i recipients. DKA and AKI were reported more frequently in adults than in older adults (P_Interaction_ < 0.05). None of the pre-specified AEs was reported more frequently in older adults.

**Fig. 3:**
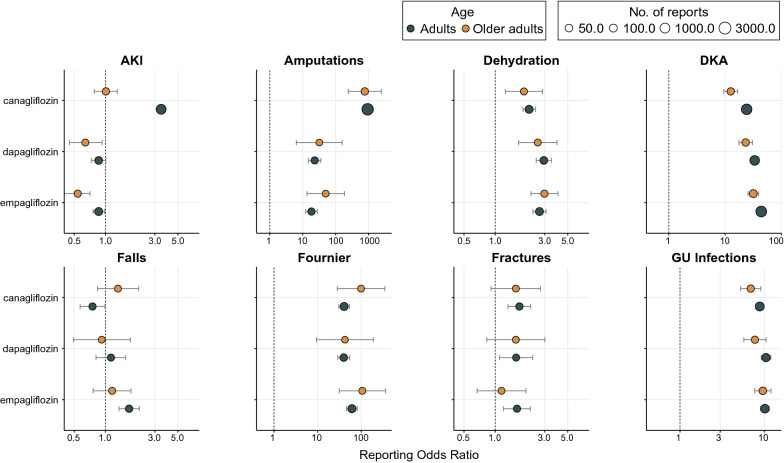
Selected safety signals stratified by SGLT2-inhibitor agent. Selected statistically significant safety signals were stratified by SGLT2-inhibitor type (canagliflozin: 10,115 adults and 757 older adults; dapagliflozin: 4,560 adults and 539 older adults; empagliflozin: 7,078 adults and 1,034 older adults). RORs of every drug-AE pair were compared to non-insulin antidiabetic drugs from the full database (88,502 adults and 17,040 older adults). A lower limit of the ROR 95% CI above 1 is the conventional threshold for significant signal detection. Adults are patients aged 18–75, older adults are 75 years or older. ROR- Reporting odds ratio; AKI- Acute kidney injury; CI- Confidence interval; DKA- Diabetic ketoacidosis; GU- genitourinary; NIAD- non-insulin antidiabetic drugs; SGLT2- Sodium-glucose co-transporter 2

In the stratified analysis by SGLT2i type, none of the AEs was more frequently reported in older adults than adults, consistent with the findings of the main analysis (Fig. 3). AKI disproportionality signal was statistically significant only in adult canagliflozin recipients, and not in older adults or recipients of other SGLT2i types. Amputations were also reported more frequently by canagliflozin recipients. All other safety signals were consistent across SGLT2i types. When AKI events were stratified by reporting year, the disproportionality signals were statistically significant between 2014 and 2017 but not in the following years (Additional file 2: Table S4).

Overall, the most frequent medications used concomitantly with SGLT2i were metformin (9,480 [39.1% of SGLT2i recipients]), statins (4,051 [16.7%]), dipeptidyl peptidase-4 inhibitors (3,661 [15.1%]), sulfonylureas (2,718 [11.2%]), ACE inhibitors (2,287 [9.4%]), and ARBs (1,971 [8.1%]) (Additional file 2: Table S5). When the reports were stratified by adverse event type, loop diuretics and thiazides were more frequently coadministered in patients who developed AKI, dehydration, falls, and hypotension. Coadministration of ACE inhibitors and ARBs was more common in patients with AKI, dehydration, hypotension, syncope, hyperkalemia, genitourinary infections, and Fournier gangrene. Combination therapy of NIAD was more common among patients who developed DKA, dehydration, and hypoglycemia (Additional file 2: Table S5).

## Discussion

As indications for SGLT2i treatment are expanding, a growing number of older adults have become candidates for treatment. Thus, characterizing treatment safety in this population is a current clinical need. In this post-marketing study, SGLT2i-related AEs were reported similarly between age groups. Amputations, Fournier gangrene, DKA, genitourinary infections, dehydration, nocturia, and fractures were over-reported in SGLT2i recipients of both age groups. These findings were in comparison to patients treated with other NIAD and were consistent across SGLT2i types (canagliflozin, empagliflozin, and dapagliflozin). Our findings provide reassurance regarding SGLT2i treatment in older adults, although careful monitoring is warranted.

Genitourinary tract infections are among the most common AEs of SGLT2i in clinical trials [25]. They are of particular interest in the older population due to increased susceptibility and potentially worse outcomes. In the FAERS, genitourinary tract infections were more commonly reported among individuals treated with SGLT2i as compared to other NIAD. However, no difference between age groups was found. These findings are in line with sub-analyses of randomized controlled trials (RCTs) which did not observe an increased risk of genitourinary tract infections in older adults compared to younger patients [8, 26, 27]. Fournier gangrene, a necrotizing soft tissue infection of the perineum, was previously reported as a potentially rare and fatal complication of SGLT2i therapy in postmarketing studies [28, 29]. Concordantly, SGLT2i were associated with increased reporting of Fournier gangrene in our study. The increased risk is apparently mediated by the increased incidence of genitourinary tract infections, which in severe cases can penetrate the urethral mucosa. As with genitourinary infections, Fournier gangrene was reported similarly in adults and older adults.

SGLT2i may increase the risk of dehydration due to their osmotic diuretic effect, particularly in older adults and patients receiving additional diuretics or antihypertensives. These, in turn, may result in falls, syncope, and fractures. In this study, we observed increased reporting of nocturia and dehydration following SGLT2i treatment in the entire cohort, without differences between age groups. Indeed, concomitant use of diuretics and hypertensives was more common among those patients. RCT sub-analyses of the older population reported mixed results regarding dehydration risk. In the EMPA-REG OUTCOME, patients older than 75 years receiving empagliflozin had an increased frequency of volume depletion, but not patients aged < 75 years [26]. In contrast, older dapagliflozin recipients had a similar frequency of dehydration to younger patients in the DECLARE–TIMI 58 and DAPA-HF studies [27, 30]. In our stratified analysis, dehydration reporting did not differ among SGLT2i types. Despite their blood pressure-lowering effect, SGLT2i treatment did not result in hypotension among the pivotal RCTs participants, including older adults. [8, 30] This finding was consistent in patients with severely reduced systolic function, who are particularly susceptible to hypotension [31]. Concordantly, SGLT2i were not associated with an increased reporting of hypotension in both age groups in the FAERS.

Fractures are another debated concern of SGLT2i treatment in the older population, which can primarily result from hypotension and falls. We found a borderline significance of increased fractures reporting, without significant differences between age groups or products. Most of the RCTs and postmarketing studies did not observe an increased incidence of fractures following SGLT2i [32, 33], apart from the CANVAS trial in which fractures were more common among canagliflozin recipients [34]. Considering the borderline significance and the lack of over-reporting of hypotension and falls in the FAERS (which are the major mediators), we do not identify a robust safety signal of fractures. Overall, in this study, we did not find an increased reporting of the major potential sequelae of volume depletion- falls, hypotension, syncope events, and fractures.

Amputations were associated with canagliflozin in clinical trials, but not empagliflozin or dapagliflozin [9, 35, 36]. Several postmarketing studies found an increased risk of amputation with all SGLT2i [11, 37], while others observed this association only for canagliflozin [38, 39]. Our analysis of contemporary postmarketing data is in line with previous studies, demonstrating increased reporting of amputations following SGLT2i treatment, particularly with canagliflozin. One population-based cohort study addressed the effect of age on amputation risk and observed an increased rate of amputations only in adults aged 65 years or older with baseline cardiovascular disease, with an event rate of 1.8 per 1,000 patients [40]. In contrast, SGLT2i-related amputations are not reported more frequently by older adults than younger patients in our FAERS analysis.

SGLT2i are associated with increased reporting of DKA in the FAERS, consistent with a meta-analysis of RCTs, which included more than 60,000 patients, and postmarketing large-scale population studies [41–44]. Although DKA-precipitating factors (e.g., intercurrent illnesses, reduced food and fluid intake) are more common among older adults, previous cohort and pharmacovigilance studies did not observe an increased risk of SGLT2i-associated DKA in this population [43, 44]. Likewise, we did not find excessive reporting of this potential life-threatening AE in older adults.

SGLT2i can cause an initial decline in the estimated glomerular filtration rate (eGFR), usually within the first months of treatment, due to intravascular volume changes [45]. This decline raised concerns regarding AKI development and its long-term outcomes, particularly in high-risk patients (e.g., elderly and patients with reduced baseline eGFR). Recently, a growing body of evidence indicates that initial eGFR decline is usually small and not associated with long-term greater eGFR decline or worse clinical outcomes [45–47]. Furthermore, several clinical trials found that SGLT2i slowed the rate of eGFR decline irrespective of diabetes [46, 48]. In the FAERS, a moderately increased reporting of AKI was found in adults but not older adults. This finding was unexpected since older adults are at a higher risk to develop AKI, and therefore we conducted post-hoc sensitivity analyses to assess for reporting bias. When AKI events were stratified by reporting year, we observed a statistically significant disproportionality signal only in the first years after approval, which has lost statistical significance since 2018. This reporting pattern may therefore reflect a notoriety bias caused by the initial concerns regarding SGLT2i-related AKI, which were mitigated with the growing understanding of the interplay between SGLT2i treatment and renal function [20]. Moreover, a high proportion of patients who reported AKI were concomitantly treated with diuretics, ACE inhibitors, or ARBs, suggesting a mutual contribution to the eGFR decline. As SGLT2i demonstrated a long-term reduction in CKD progression in various trials [10, 46, 48], understanding the temporary nature of eGFR decline is important to avoid the discontinuation of SGLT2i in the older population.

## Limitations

Several limitations of this pharmacovigilance study should be acknowledged. First, this study provides absolute numbers and disproportionality measures but not a true incidence, which cannot be calculated using the FAERS since the number of patients exposed to the drugs is unknown. Therefore, although we did not find significant differences in the reporting of SGLT2i-related AEs between adults and older adults, the incidence rate of these AEs might still be higher in the older population. Second, granular data on patients’ characteristics, comorbidities, and diabetic complications are lacking in the FAERS. To mitigate potential confounders, we included only patients with T2DM and used a restricted comparator group of NIAD other than SGLT2i, which represents a population sharing common features with SGLT2i recipients. Third, concomitant drugs may have a contributive role in the AEs occurrence. Although older adults were treated more frequently with various concomitant medications (e.g., diuretics, anti-hypertensives, and statins), we did not find differences in the AEs reporting between age groups. These findings underscore the external validity of our analysis which represents real-world settings. Finally, lack of statistical power is an inherent limitation of sub-group analysis, particularly when analyzing infrequent AEs, such as DKA and Fournier gangrene. However, the FAERS provides an opportunity to address this limitation due to its large sample size and longer follow-up compared to clinical trials. Clinical trials may also not reflect the real-world population, particularly the older population which is underrepresented. Furthermore, some of the AEs, such as DKA, are less likely to occur in closely monitored participants in the RCT setting compared to a real-world setting. Therefore, global post-marketing surveillance programs can provide essential complementary information.

## Conclusions and clinical implications

In this post-marketing study, none of the pre-specified AEs was reported more frequently by older adult recipients of SGLT2i as compared to younger recipients. Amputations, Fournier gangrene, DKA, genitourinary infections, dehydration, nocturia, and fractures were overreported in both age groups in contemporary post-marketing surveillance data, and therefore still represent treatment safety concerns. Overall, our findings support the evidence-based utilization of SGLT2i in older adults, although careful monitoring is warranted.

## Supplementary Information

**Additional file 1: Table S1.** Adverse events grouping by the Medical Dictionary for Regulatory Activities (MedDRA version 25.1) classification.

**Additional file 2: Table S2.** Demographic and clinical characteristics of patients treated with non-insulin antidiabetics (NIAD) in the FAERS. **Table S3.** Unadjusted disproportionality analysis of SGLT2-inhibitors-related adverse events compared to other non-insulin anti-diabetics. **Table S4.** Absolute numbers and RORs of SGLT2-inhibitors-related AKI by year. **Table S5.** The most frequently reported concomitant drugs by adverse event (AE) type.

## Data Availability

The dataset supporting the conclusions of this article is available in the FDA Adverse Event Reporting System (FAERS) Quarterly Data Files, at https://fis.fda.gov/extensions/FPD-QDE-FAERS/FPD-QDE-FAERS.html.
