# Supplementary material for: The real-world safety profile of sodium-glucose co-transporter-2 inhibitors among older adults (≥ 75 years): a retrospective, pharmacovigilance study
Source: Cardiovasc Diabetol. 2023 Jan 24;22:16. doi: 10.1186/s12933-023-01743-5 (PMC9875397; doi:10.1186/s12933-023-01743-5)
Supplement: Supplementary file 2 — Additional file 2: Table S2. Demographic and clinical characteristics of patients treated with non-insulin antidiabetics (NIAD) in the FAERS. Table S3. Unadjusted disproportionality analysis of SGLT2-inhibitors-related adverse events compared to other non-insulin anti-diabetics. Table S4. Absolute numbers and RORs of SGLT2-inhibitors-related AKI by year. Table S5. The most frequently reported concomitant drugs by adverse event (AE) type. [file 12933_2023_1743_MOESM2_ESM.docx]

Additional file 1: Table S2: Demographic and clinical characteristics of patients treated with non-insulin antidiabetics (NIAD) in the FAERS

|  | **ALL (129,795)** | **SGLT2 inhibitors (24,253)** | **Other NIAD (105,542)** |
| --- | --- | --- | --- |
| **Region** |  | | |
| America | 95,131/126,911 (75.0) | 16,427/23,656 (69.4) | 78,704/103,245 (76.2) |
| Europe | 19,157/126,911 (15.1) | 3,972/23,656 (16.7) | 15,185/103,245 (14.8) |
| Asia | 11,281/126,911 (8.9) | 2,758/23,656 (11.6) | 8,513/103,245 (8.2) |
| Africa | 295/126,911 (0.2) | 61/23,656 (0.3) | 234/103,245 (0.2) |
| Australia | 1,047/126,911 (0.8) | 438/23,656 (2.0) | 609/103,245 (0.6) |
| **Reporter** |  | | |
| Health professional | 68,821/122,903 (55.9) | 12,882/23,100 (55.8) | 55,939/99,803 (56.0) |
| Patient/lawyer | 54,082/122,903 (44.1) | 10,218/23,100 (44.2) | 43,864 /2,222 (44.0) |
| **Year of report** |  | | |
| 2014^a^ | 10,374/129,795 (8.0) | 652/24,253 (2.7) | 9,722/105,542 (9.2) |
| 2015 | 32,346/129,795 (24.9) | 3,465/24,253 (14.3) | 28,881/105,542 (27.4) |
| 2016 | 15,829/129,795 (12.2) | 2,747/24,253 (11.3) | 13,082/105,542 (12.4) |
| 2017 | 13,426/129,795 (10.3) | 3,057/24,253 (12.6) | 10,369/105,542 (9.8) |
| 2018 | 17,818/129,795(13.7) | 5,426/24,253 (22.4) | 12,392/105,542 (11.7) |
| 2019 | 15,293/129,795 (11.8) | 4,038/24,253 (16.6) | 11,255/105,542 (10.7) |
| 2020 | 16,273/129,795 (12.5) | 3,201/24,253 (13.2) | 13,072/105,542 (12.4) |
| 2021 | 8,436/129,795 (6.5) | 1,667/24,253 (6.9) | 6,769/105,542 (6.4) |
| **Age group** |  | | |
| Adults | 110,416/129,795 (85.1) | 21,914/24,253 (90.3) | 88,502/105,542 (83.9) |
| Older Adults | 19,379/129,795 (14.9) | 2,339/24,253 (9.6) | 17,040/105,542 (16.1) |
| **Age**, years |  | | |
| Median (IQR) | 63 (53-72) | 60 (51-68) | 64 (54-73) |
| **Sex** |  | | |
| Females | 64,963/127,902 (50.8) | 10,885/24,006 (45.3) | 54,078/103,896 (52.1) |
| Males | 62,939/127,902 (49.2) | 13,121/24,006 (54.7) | 49,818/103,896 (47.9) |

Values are n/N (%) unless otherwise specified.

1. Americas category includes north and south America; Out of 95,131 patients from the Americas in the entire cohort, 90,232 were from the United States (15,158 recipients of SGLT2 inhibitors and 75,074 of other NIAD).
2. Only reports from July to December were included.

Abbreviations: FAERS- FDA adverse event reporting system; IQR- Interquartile range; SGLT2- sodium-glucose co-transporter-2.

Additional file 1: Table S3: Unadjusted disproportionality analysis of SGLT2-inhibitors-related adverse events compared to other non-insulin anti-diabetics.

|  | Adults (< 75 years) | | | Older adults (≥75 years) | | |  |
| --- | --- | --- | --- | --- | --- | --- | --- |
| Adverse event | Total reports, n | ROR | IC_025_ | Total reports, n | ROR | IC_025_ | |
| AKI | 1758 | 1.99 (1.87-2.11) | 0.62 | 147 | 0.71 (0.59-0.84) | -0.66 | |
| Amputations | 3054 | 367.30 (267.72-503.92) | 2.26 | 102 | 258.92 (82.07-816.90) | 2.67 | |
| Dehydration | 643 | 2.46 (2.23-2.72) | 0.80 | 102 | 2.52 (2.00-3.17) | 0.76 | |
| DKA | 5226 | 31.80 (29.54-34.24) | 2.07 | 399 | 22.85 (18.85-27.71) | 2.43 | |
| Falls | 179 | 1.13 (0.96-1.34) | -0.08 | 54 | 1.15 (0.86-1.54) | -0.24 | |
| Fournier gangrene | 666 | 46.99 (35.99-61.34) | 2.09 | 36 | 88.77 (27.31-288.48) | 2.30 | |
| Fractures | 155 | 1.65 (1.37-1.99) | 0.31 | 38 | 1.38 (0.98-1.96) | -0.10 | |
| Genitourinary infections | 1580 | 9.66 (8.83-10.57) | 1.73 | 258 | 8.29 (6.93-9.92) | 1.88 | |
| Hyperkalemia | 161 | 1.19 (0.99-1.41) | -0.04 | 20 | 0.62 (0.39-0.98) | -1.30 | |
| Hypoglycemia | 228 | 0.25 (0.22-0.29) | -1.89 | 43 | 0.19 (0.14-0.26) | -2.57 | |
| Hypotension | 312 | 1.06 (0.93-1.20) | -0.10 | 46 | 1.29 (0.94-1.77) | -0.14 | |
| Nocturia | 58 | 5.73 (3.84-8.54) | 1.14 | 10 | 6.65 (2.82-15.67) | 0.76 | |
| Syncope | 199 | 1.14 (0.98-1.34) | -0.06 | 38 | 1.12 (0.79-1.58) | -0.36 | |

Disproportionality analysis of SGLT2-inhibitors-related adverse events as compared to other non-insulin antidiabetic drugs from the full database. A lower bound of the ROR 95% confidence interval above one and a positive IC_025_ value are the conventional thresholds for significant signal detection. Adults are patients aged 18-75, older adults are 75 years or older.

Abbreviations: AKI- Acute kidney injury; DKA- Diabetic ketoacidosis; IC- information component; ROR- reporting odds ratio; SGLT2- Sodium-glucose co-transporter 2.

Additional file 1: Table S4: Absolute numbers and RORs of SGLT2-inhibitors-related AKI by year.

| **Year** | **Total SGLT2-inhibitors reports,** n^a^ | **SGLT2-inhibitors-related AKI reports,** n (%)^b^ | **ROR**^c^ (95% CI) |
| --- | --- | --- | --- |
| 2014 | 1585 | 214 (13.5) | 2.73 (2.25-3.32) |
| 2015 | 3587 | 616 (17.2) | 4.54 (3.92-5.26) |
| 2016 | 3501 | 414 (11.8) | 3.91 (3.35-4.55) |
| 2017 | 3071 | 173 (5.6) | 1.40 (1.16-1.70) |
| 2018 | 2106 | 95 (4.5) | 1.11 (0.88-1.41) |
| 2019 | 1894 | 71 (3.7) | 0.84 (0.64-1.09) |
| 2020 | 1817 | 64 (3.5) | 1.23 (0.92-1.64) |
| 2021 | 668 | 16 (2.4) | 0.97 (0.55-1.70) |

1. The total number of SGLT2-inhibitors reports by year of reporting.
2. The number of SGLT2-inhibitors-related AKI cases by year of reporting and their proportion (%) out of the total number of SGLT2-inhibitors reports in the corresponding year.
3. The ROR of SGLT2-inhibitors-related AKI compared to other NIAD in each year.

Abbreviations: AKI- acute kidney injury, ROR- reporting odds ratio; SGLT2- sodium-glucose co-transporter-2.

Additional file 1: Table S5: The most frequently reported concomitant drugs by adverse event (AE) type

| **Drug name** | **Total Reports,**  **n/N (%)** | **AE** | **Reports by AE type,**  **n/N (%)** | **P Val** |
| --- | --- | --- | --- | --- |
| Biguanides | 9480/24253 (39.09%) | AKI | 764/1905 (40.10%) | 0.372 |
| Statins | 4051/24253 (16.70%) | AKI | 236/1905 (12.39%) | <0.001 |
| DPP-4 inhibitors | 3661/24253 (15.10%) | AKI | 223/1905 (11.71%) | <0.001 |
| ACE inhibitors | 2287/24253 (9.43%) | AKI | 210/1905 (11.02%) | 0.019 |
| Sulfonylureas | 2718/24253 (11.21%) | AKI | 175/1905 (9.19%) | 0.005 |
| ARBs | 1971/24253 (8.13%) | AKI | 126/1905 (6.61%) | 0.015 |
| Selective BB | 1432/24253 (5.90%) | AKI | 110/1905 (5.77%) | 0.846 |
| GLP-1 analogues | 1370/24253 (5.65%) | AKI | 92/1905 (4.83%) | 0.124 |
| DHP CCB | 1549/24253 (6.39%) | AKI | 91/1905 (4.78%) | 0.003 |
| Thiazides | 863/24253 (3.56%) | AKI | 86/1905 (4.51%) | 0.030 |
| Biguanides | 9480/24253 (39.09%) | Amputations | 445/3156 (14.10%) | <0.001 |
| Sulfonylureas | 2718/24253 (11.21%) | Amputations | 66/3156 (2.09%) | <0.001 |
| DPP-4 inhibitors | 3661/24253 (15.10%) | Amputations | 64/3156 (2.03%) | <0.001 |
| ACE inhibitors | 2287/24253 (9.43%) | Amputations | 41/3156 (1.30%) | <0.001 |
| Statins | 4051/24253 (16.70%) | Amputations | 41/3156 (1.30%) | <0.001 |
| GLP-1 analogues | 1370/24253 (5.65%) | Amputations | 24/3156 (0.76%) | <0.001 |
| ARBs | 1971/24253 (8.13%) | Amputations | 19/3156 (0.60%) | <0.001 |
| DHP CCB | 1549/24253 (6.39%) | Amputations | 18/3156 (0.57%) | <0.001 |
| Selective BB | 1432/24253 (5.90%) | Amputations | 17/3156 (0.54%) | <0.001 |
| Sulfonamides | 574/24253 (2.37%) | Amputations | 16/3156 (0.51%) | <0.001 |
| Biguanides | 9480/24253 (39.09%) | Dehydration | 330/745 (44.30%) | 0.004 |
| Statins | 4051/24253 (16.70%) | Dehydration | 152/745 (20.40%) | 0.008 |
| DPP-4 inhibitors | 3661/24253 (15.10%) | Dehydration | 151/745 (20.27%) | <0.001 |
| Sulfonylureas | 2718/24253 (11.21%) | Dehydration | 100/745 (13.42%) | 0.063 |
| ACE inhibitors | 2287/24253 (9.43%) | Dehydration | 90/745 (12.08%) | 0.017 |
| ARBs | 1971/24253 (8.13%) | Dehydration | 84/745 (11.28%) | 0.003 |
| Selective BB | 1432/24253 (5.90%) | Dehydration | 62/745 (8.32%) | 0.008 |
| GLP-1 analogues | 1370/24253 (5.65%) | Dehydration | 62/745 (8.32%) | 0.003 |
| PPI | 1518/24253 (6.26%) | Dehydration | 61/745 (8.19%) | 0.034 |
| DHP CCB | 1549/24253 (6.39%) | Dehydration | 56/745 (7.52%) | 0.203 |
| Biguanides | 9480/24253 (39.09%) | DKA | 2832/5625 (50.35%) | <0.001 |
| DPP-4 inhibitors | 3661/24253 (15.10%) | DKA | 982/5625 (17.46%) | <0.001 |
| Statins | 4051/24253 (16.70%) | DKA | 928/5625 (16.50%) | 0.694 |
| Sulfonylureas | 2718/24253 (11.21%) | DKA | 670/5625 (11.91%) | 0.095 |
| ACE inhibitors | 2287/24253 (9.43%) | DKA | 482/5625 (8.57%) | 0.027 |
| GLP-1 analogues | 1370/24253 (5.65%) | DKA | 421/5625 (7.48%) | <0.001 |
| PPI | 1518/24253 (6.26%) | DKA | 369/5625 (6.56%) | 0.349 |
| ARBs | 1971/24253 (8.13%) | DKA | 283/5625 (5.03%) | <0.001 |
| DHP CCB | 1549/24253 (6.39%) | DKA | 262/5625 (4.66%) | <0.001 |
| Selective BB | 1432/24253 (5.90%) | DKA | 235/5625 (4.18%) | <0.001 |
| Biguanides | 9480/24253 (39.09%) | Falls | 102/233 (43.78%) | 0.158 |
| DPP-4 inhibitors | 3661/24253 (15.10%) | Falls | 54/233 (23.18%) | 0.001 |
| Statins | 4051/24253 (16.70%) | Falls | 46/233 (19.74%) | 0.219 |
| Sulfonylureas | 2718/24253 (11.21%) | Falls | 34/233 (14.59%) | 0.118 |
| ACE inhibitors | 2287/24253 (9.43%) | Falls | 25/233 (10.73%) | 0.500 |
| ARBs | 1971/24253 (8.13%) | Falls | 22/233 (9.44%) | 0.471 |
| PPI | 1518/24253 (6.26%) | Falls | 22/233 (9.44%) | 0.056 |
| DHP CCB | 1549/24253 (6.39%) | Falls | 21/233 (9.01%) | 0.107 |
| GLP-1 analogues | 1370/24253 (5.65%) | Falls | 17/233 (7.30%) | 0.256 |
| Sulfonamides | 574/24253 (2.37%) | Falls | 16/233 (6.87%) | <0.001 |
| Biguanides | 9480/24253 (39.09%) | Fournier gangrene | 265/702 (37.75%) | 0.486 |
| Statins | 4051/24253 (16.70%) | Fournier gangrene | 127/702 (18.09%) | 0.336 |
| ACE inhibitors | 2287/24253 (9.43%) | Fournier gangrene | 101/702 (14.39%) | <0.001 |
| DPP-4 inhibitors | 3661/24253 (15.10%) | Fournier gangrene | 87/702 (12.39%) | 0.045 |
| Sulfonylureas | 2718/24253 (11.21%) | Fournier gangrene | 61/702 (8.69%) | 0.036 |
| PPI | 1518/24253 (6.26%) | Fournier gangrene | 59/702 (8.40%) | 0.023 |
| Selective BB | 1432/24253 (5.90%) | Fournier gangrene | 52/702 (7.41%) | 0.093 |
| ARBs | 1971/24253 (8.13%) | Fournier gangrene | 46/702 (6.55%) | 0.146 |
| GLP-1 analogues | 1370/24253 (5.65%) | Fournier gangrene | 46/702 (6.55%) | 0.288 |
| Natural opium alkaloids | 268/24253 (1.11%) | Fournier gangrene | 39/702 (5.56%) | <0.001 |
| Biguanides | 9480/24253 (39.09%) | Fractures | 85/193 (44.04%) | 0.162 |
| DPP-4 inhibitors | 3661/24253 (15.10%) | Fractures | 42/193 (21.76%) | 0.015 |
| Statins | 4051/24253 (16.70%) | Fractures | 32/193 (16.58%) | >0.99 |
| Sulfonylureas | 2718/24253 (11.21%) | Fractures | 30/193 (15.54%) | 0.067 |
| DHP CCB | 1549/24253 (6.39%) | Fractures | 23/193 (11.92%) | 0.004 |
| ARBs | 1971/24253 (8.13%) | Fractures | 20/193 (10.36%) | 0.237 |
| PPI | 1518/24253 (6.26%) | Fractures | 18/193 (9.33%) | 0.099 |
| ACE inhibitors | 2287/24253 (9.43%) | Fractures | 16/193 (8.29%) | 0.711 |
| GLP-1 analogues | 1370/24253 (5.65%) | Fractures | 13/193 (6.74%) | 0.530 |
| Selective BB | 1432/24253 (5.90%) | Fractures | 12/193 (6.22%) | 0.761 |
| Biguanides | 9480/24253 (39.09%) | GU infections | 750/1838 (40.81%) | 0.132 |
| Statins | 4051/24253 (16.70%) | GU infections | 347/1838 (18.88%) | 0.013 |
| DPP-4 inhibitors | 3661/24253 (15.10%) | GU infections | 298/1838 (16.21%) | 0.182 |
| Sulfonylureas | 2718/24253 (11.21%) | GU infections | 248/1838 (13.49%) | 0.002 |
| ACE inhibitors | 2287/24253 (9.43%) | GU infections | 188/1838 (10.23%) | 0.247 |
| ARBs | 1971/24253 (8.13%) | GU infections | 183/1838 (9.96%) | 0.005 |
| Selective BB | 1432/24253 (5.90%) | GU infections | 128/1838 (6.96%) | 0.060 |
| DHP CCB | 1549/24253 (6.39%) | GU infections | 127/1838 (6.91%) | 0.365 |
| PPI | 1518/24253 (6.26%) | GU infections | 124/1838 (6.75%) | 0.386 |
| GLP-1 analogues | 1370/24253 (5.65%) | GU infections | 93/1838 (5.06%) | 0.289 |
| Biguanides | 9480/24253 (39.09%) | Hyperkalemia | 71/181 (39.23%) | >0.99 |
| ACE inhibitors | 2287/24253 (9.43%) | Hyperkalemia | 43/181 (23.76%) | <0.001 |
| Statins | 4051/24253 (16.70%) | Hyperkalemia | 33/181 (18.23%) | 0.551 |
| DPP-4 inhibitors | 3661/24253 (15.10%) | Hyperkalemia | 27/181 (14.92%) | >0.99 |
| Selective BB | 1432/24253 (5.90%) | Hyperkalemia | 19/181 (10.50%) | 0.016 |
| Sulfonylureas | 2718/24253 (11.21%) | Hyperkalemia | 18/181 (9.94%) | 0.723 |
| ARBs | 1971/24253 (8.13%) | Hyperkalemia | 15/181 (8.29%) | 0.892 |
| DHP CCB | 1549/24253 (6.39%) | Hyperkalemia | 14/181 (7.73%) | 0.445 |
| Aldosterone antagonists | 255/24253 (1.05%) | Hyperkalemia | 13/181 (7.18%) | <0.001 |
| PPI | 1518/24253 (6.26%) | Hyperkalemia | 12/181 (6.63%) | 0.759 |
| Biguanides | 9480/24253 (39.09%) | Hypoglycemia | 144/271 (53.14%) | <0.001 |
| DPP-4 inhibitors | 3661/24253 (15.10%) | Hypoglycemia | 67/271 (24.72%) | <0.001 |
| Sulfonylureas | 2718/24253 (11.21%) | Hypoglycemia | 55/271 (20.30%) | <0.001 |
| Statins | 4051/24253 (16.70%) | Hypoglycemia | 50/271 (18.45%) | 0.463 |
| ARBs | 1971/24253 (8.13%) | Hypoglycemia | 29/271 (10.70%) | 0.120 |
| GLP-1 analogues | 1370/24253 (5.65%) | Hypoglycemia | 25/271 (9.23%) | 0.017 |
| ACE inhibitors | 2287/24253 (9.43%) | Hypoglycemia | 24/271 (8.86%) | 0.835 |
| Selective BB | 1432/24253 (5.90%) | Hypoglycemia | 22/271 (8.12%) | 0.122 |
| DHP CCB | 1549/24253 (6.39%) | Hypoglycemia | 20/271 (7.38%) | 0.457 |
| Alpha glucosidase inhibitors | 284/24253 (1.17%) | Hypoglycemia | 14/271 (5.17%) | <0.001 |
| Biguanides | 9480/24253 (39.09%) | Hypotension | 153/358 (42.74%) | 0.159 |
| ACE inhibitors | 2287/24253 (9.43%) | Hypotension | 70/358 (19.55%) | <0.001 |
| Statins | 4051/24253 (16.70%) | Hypotension | 64/358 (17.88%) | 0.571 |
| ARBs | 1971/24253 (8.13%) | Hypotension | 52/358 (14.53%) | <0.001 |
| Sulfonylureas | 2718/24253 (11.21%) | Hypotension | 48/358 (13.41%) | 0.181 |
| Selective BB | 1432/24253 (5.90%) | Hypotension | 46/358 (12.85%) | <0.001 |
| DPP-4 inhibitors | 3661/24253 (15.10%) | Hypotension | 42/358 (11.73%) | 0.077 |
| DHP CCB | 1549/24253 (6.39%) | Hypotension | 30/358 (8.38%) | 0.129 |
| Thiazides | 863/24253 (3.56%) | Hypotension | 30/358 (8.38%) | <0.001 |
| GLP-1 analogues | 1370/24253 (5.65%) | Hypotension | 28/358 (7.82%) | 0.085 |
| Biguanides | 9480/24253 (39.09%) | Nocturia | 36/68 (52.94%) | 0.025 |
| Statins | 4051/24253 (16.70%) | Nocturia | 17/68 (25.00%) | 0.073 |
| ARBs | 1971/24253 (8.13%) | Nocturia | 12/68 (17.65%) | 0.011 |
| PPI | 1518/24253 (6.26%) | Nocturia | 10/68 (14.71%) | 0.010 |
| DHP CCB | 1549/24253 (6.39%) | Nocturia | 9/68 (13.24%) | 0.040 |
| DPP-4 inhibitors | 3661/24253 (15.10%) | Nocturia | 9/68 (13.24%) | 0.865 |
| Sulfonylureas | 2718/24253 (11.21%) | Nocturia | 9/68 (13.24%) | 0.564 |
| ACE inhibitors | 2287/24253 (9.43%) | Nocturia | 8/68 (11.76%) | 0.530 |
| Alpha-adrenoreceptor antagonists | 345/24253 (1.42%) | Nocturia | 6/68 (8.82%) | <0.001 |
| Selective BB | 1432/24253 (5.90%) | Nocturia | 6/68 (8.82%) | 0.296 |
| Biguanides | 9480/24253 (39.09%) | Syncope | 100/237 (42.19%) | 0.351 |
| Statins | 4051/24253 (16.70%) | Syncope | 51/237 (21.52%) | 0.055 |
| DPP-4 inhibitors | 3661/24253 (15.10%) | Syncope | 43/237 (18.14%) | 0.203 |
| ACE inhibitors | 2287/24253 (9.43%) | Syncope | 36/237 (15.19%) | 0.005 |
| Sulfonylureas | 2718/24253 (11.21%) | Syncope | 33/237 (13.92%) | 0.181 |
| ARBs | 1971/24253 (8.13%) | Syncope | 25/237 (10.55%) | 0.189 |
| Selective BB | 1432/24253 (5.90%) | Syncope | 25/237 (10.55%) | 0.005 |
| GLP-1 analogues | 1370/24253 (5.65%) | Syncope | 17/237 (7.17%) | 0.322 |
| PPI | 1518/24253 (6.26%) | Syncope | 17/237 (7.17%) | 0.504 |
| DHP CCB | 1549/24253 (6.39%) | Syncope | 16/237 (6.75%) | 0.790 |

The table presents the medications that were most frequently reported concomitantly with SGLT2 inhibitors overall and among patients with each of the adverse events. P values were calculated by the binomial test for the proportion of patients using the drug among those who developed the adverse event compared to the corresponding proportion in the entire cohort.

Abbreviations: ACE inhibitors- angiotensin-converting enzyme inhibitors; AKI- acute kidney injury; ARBs- angiotensin II receptor blockers; BB- beta-blockers; DHP CCB- dihydropyridine calcium channel blockers; DKA- diabetic ketoacidosis; DPP-4- dipeptidyl peptidase-4; GLP1- glucagon-like peptide 1; GU- genitourinary; PPI- proton pump inhibitors; SGLT2- sodium-glucose co-transporter-2.
